# The Digital Amplifier in Medical Insurance: How Chinese Provincial Pooling Policy Optimizes Chronic Disease Management

**DOI:** 10.3390/healthcare13202643

**Published:** 2025-10-21

**Authors:** Ming Zeng, Huan Cheng, Weike Zhang

**Affiliations:** 1 School of Economics, Xihua University, Chengdu 610039, China; zengming@mail.xhu.edu.cn; 2 School of Public Administration, Sichuan University, Chengdu 610065, China; zhangwk@scu.edu.cn

**Keywords:** chronic disease management, provincial pooling, digital technology, health outcome, Basic Medical Insurance Program (BMIP)

## Abstract

**Background:** Chronic diseases have proliferated worldwide and become one of the foremost public health challenges. The provincial pooling policy of Chinese Basic Medical Insurance Program (BMIP) (hereinafter the Policy) is conducive to coordinating healthcare resources more broadly and containing medical costs more effectively, which creates opportunities to improve chronic disease patients’ health outcomes. Against this backdrop, this study aims to identify how the Policy affects chronic disease patients’ health outcomes. **Methodology:** Utilizing data from the China Family Panel Studies (CFPS) across 31 provinces (except Hong Kong, Macao, and Taiwan) from 2010 to 2022, we constructed a panel of 26,585 observations on chronic disease patients enrolled in the BMIP. We employed a difference-in-differences (DID) design to identify the causal effects of the Policy on self-rated health (SRH) supplemented by a series of robustness checks, including event-study analysis, placebo tests, and propensity score matching DID (PSM-DID). **Results:** The results show that the Policy enhances Chinese chronic disease patients’ health outcomes across various robustness assessments. However, the effects exhibit heterogeneity in that the Policy can more effectively improve the health outcomes of urban patients, low-income patients, and highly educated patients. The mechanism analysis indicates that the Policy can enhance chronic disease patients’ health outcomes by reducing the out-of-pocket ratio, increasing household income, and stimulating consumer expenditure. Furthermore, digital technology can amplify the effectiveness of the Policy in Chinese chronic disease patients’ health outcomes. **Conclusions:** These findings provide valuable insights into the potential of provincial pooling and digital technology to optimize Chinese chronic disease management.

## 1. Introduction

Against the backdrop of the sustained rise in global life expectancy, pervasive unhealthy lifestyles, and persistent environmental degradation, chronic diseases have proliferated worldwide, emerging as one of the foremost public health challenges of the 21st century [1,2]. In particular, cardiovascular diseases, cancer, diabetes, and chronic respiratory diseases—which constitute the core of the chronic disease burden and represent the predominant forms of non-communicable diseases (NCDs)—are regarded as the “central challenge” and “top priority” in chronic disease management [3]. According to World Health Statistics 2025 issued by the World Health Organization (WHO) (World health statistics 2025: monitoring health for the SDGs, sustainable development goals, https://www.who.int/publications/i/item/9789240110496/ (accessed on 25 August 2025)), premature deaths from NCDs (in individuals under 70 years of age) reached 18 million globally in 2021, accounting for over half of all deaths in this category. Furthermore, the WHO estimated that in 2019, a 30-year-old individual had an 18% probability of premature death from one of these four major chronic diseases. Chronic diseases pose an especially severe threat to developing countries, primarily attributable to the limited availability of essential healthcare resources, as well as dietary imbalances and adverse environmental changes associated with rapid urbanization [4]. China is experiencing a rising prevalence of chronic diseases, which represent more than 80% of all causes of death in its population (Proceedings from the “Promoting High-Quality Development” Press Conference Series, convened by the State Council Information Office (National Health Commission), https://www.nhc.gov.cn/xcs/c100122/202409/ca93d6c8ae9e45a0a94e1b2657e60f2d.shtml (accessed on 25 August 2025)). The Chinese government has implemented the “Healthy China” strategy, steadily advancing the whole-process management of chronic diseases encompassing prevention, screening, treatment, and rehabilitation, and is committed to improving population health outcomes and reducing premature mortality caused by major chronic diseases [5,6,7].

Unlike acute conditions, chronic diseases are characterized by elevated prevalence, increased risk of complications, prolonged treatment duration, and substantial medical expenses [8]. These attributes necessitate a systematic, continuous, and integrated management model [9]. Such an approach requires cross-tier integration of healthcare resources and strengthened collaboration among medical institutions, pharmaceutical suppliers, and medical insurance agencies [6,10]. Simultaneously, chronic disease management must remain patient-centered, focusing on reducing financial burdens, enhancing health literacy, and fostering proactive disease self-management [11]. Currently, the pooling level of the Chinese Basic Medical Insurance Program (BMIP) remains predominantly at the municipal level. This fragmented pooling structure impairs risk-pooling capacity, restricts systemic resource integration, and thus fails to meet the requirements of holistic chronic disease care [12]. Advancing provincial pooling is essential to coordinate healthcare resources more broadly and contain medical costs more effectively—both critical to improving chronic disease management outcomes [13]. By 2022 (this study’s sample cutoff), nine provinces had implemented the provincial pooling policy of the Chinese BMIP (hereinafter the Policy) [8]. However, to date, no studies have been identified to investigate whether the Policy has exerted a positive impact on health outcomes among patients with chronic diseases.

A growing body of research emphasizes the critical role of social factors in chronic disease management, identifying them as crucial for improving health outcomes [14]. Studies show that individuals with lower economic status face a higher risk of developing chronic conditions compared to those with higher incomes [15]. Improving economic conditions and adopting health-promoting lifestyles may therefore help reduce the incidence of such diseases [16]. At the same time, enhancing patients’ digital literacy and technological competence can improve self-management efficacy and health outcomes in chronic care [17,18]. Moreover, medical insurance serves as a key factor in chronic disease management, since enrollment improves access to higher-quality care [19] while reducing financial barriers [20], thereby promoting better health outcomes [21].

Several studies have specifically examined the health effects of the Policy. For example, Dong (2023) [13] shows that the Policy improves enrollees’ health by alleviating financial burdens and increasing healthcare utilization. Peng et al. (2025) [22], focusing on the elderly, find that the Policy raises total medical spending and lowers out-of-pocket costs, resulting in net health benefits. Similarly, Zhang and Chen (2024) [8], studying migrant populations, show that the Policy enhances health through improved social integration. However, existing research still cannot answer two pressing questions: Can the Policy actually improve health outcomes among patients with chronic diseases? And through what specific mechanisms might such improvement occur?

**Figure 1:**
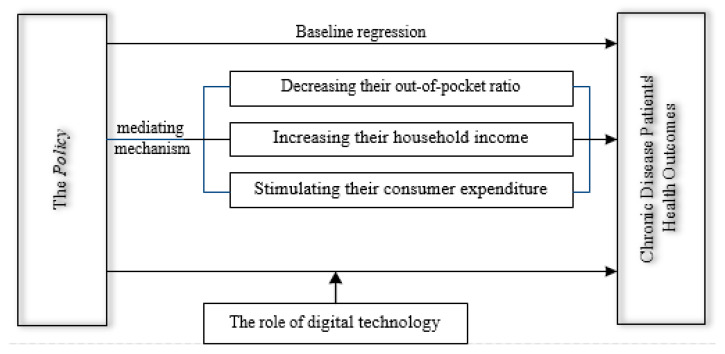
Conceptual framework.

Against this backdrop, this study identified how the Policy affects chronic disease patients’ health outcomes utilizing a total of 26,585 observations that encompassed 31 provinces in China (except Hong Kong, Macao, and Taiwan) over the period from 2010 to 2022. Subsequently, we analyzed the heterogeneity of the Policy’s health effects by examining the regional distribution, income levels, and educational attainment among chronic disease patients. We also analyzed the mechanisms through which the Policy influences chronic disease patients’ health outcomes, focusing on factors such as the out-of-pocket ratio, household income, and consumer expenditure. This study further identified how digital technology enhances the Policy’s effectiveness in improving health outcomes of chronic disease patients. Figure 1 shows the conceptual framework of this study.

## 2. Policy Background and Hypotheses

### 2.1. Policy Background

Currently, China’s BMIP operates predominantly under city pooling, wherein funds are managed uniformly within municipal jurisdictions. This decentralized approach fragments risk pools, critically undermining the system’s fiscal resilience and resulting in divergent fund performances: excessive surpluses in some regions coexisting with unsustainable deficits in others, thereby jeopardizing the BMIP’s long-term viability. To advance systemic equity and sustainability, the Chinese government has instituted provincial pooling, mandating unified fund management at the provincial tier. The Policy is anchored in the 2011 Social Insurance Law, which prescribes gradual provincial pooling implementation, and is further codified in the 2020 Central Committee/State Council Opinions on Deepening Medical Security System Reform, emphasizing provincial pooling as essential for achieving balanced, comprehensive system development.

Core components of the Policy encompass: (1) centralized fund governance, requiring provincial budget consolidation (the risk adjustment fund model is permitted as a provisional measure during the transition period); (2) standardized reimbursement protocols, harmonizing benefit parameters (ratios, drug formularies, caps) province-wide under the “highest-standard integration” principle; (3) concomitant supply-side reforms, synchronizing payment method innovations and medical service system restructuring; and (4) provincial digital integration, establishing unified information platforms for enhanced oversight efficiency. Collectively, the Policy drives China’s transition from a fragmented to integrated BMIP, advancing population-wide health improvement objectives.

### 2.2. Theoretical Analysis and Hypotheses

The well-established Andersen behavioral model identifies medical insurance systems as a critical enabling resource that fundamentally shapes healthcare utilization and health outcomes [23]. In the Chinese context, the Policy can significantly enhance chronic disease patients’ health outcomes. Firstly, the Policy facilitates the stabilization of chronic disease patients’ treatment expectations, thereby supporting their access to long-term continuous treatment [13]. By consolidating fragmented municipal insurance funds into a unified provincial pool, the Policy substantially strengthens systemic sustainability and risk-pooling capacity [24]. This fiscal integration stabilizes patients’ long-term reimbursement expectations—particularly critical for chronic diseases requiring lifelong, uninterrupted management—thereby strengthening their confidence in treatment and reducing the risk of treatment discontinuation.

Secondly, the Policy effectively reduces the medical treatment costs of patients with chronic diseases while improving their access to healthcare services and utilization of medications [22]. Managing chronic diseases is inherently complex, encompassing various aspects such as pharmacological treatment, diagnostic examinations, and management of complications, which collectively impose substantial financial burdens on patients [25]. Through the “highest-standard integration” principle, provincial pooling harmonizes previously divergent municipal insurance policies, leading to increased reimbursement rates, expanded coverage for essential drugs and services, and higher payment ceilings—all of which directly alleviate the financial burden on households. Consolidating multiple fragmented municipal medical insurance funds into a unified provincial fund enhances the bargaining power of medical insurance institutions, enabling them to reduce the costs of medical goods and services [24]. These collectively reduce medical burdens on chronic disease patients and improve their health outcomes.

Finally, the Policy fundamentally strengthens treatment adherence through standardized medical insurance policies—an essential element in chronic disease management success. The standardization of insurance rules across regions brought by the Policy eliminates treatment disruptions previously caused by cross-jurisdictional policy inconsistencies (e.g., forced “doctor-shopping” for optimal benefits) [26]. Additionally, the Policy promotes the redistribution of medical resources toward primary healthcare institutions, strengthens the service capacity of local medical facilities, reduces the unnecessary concentration of chronic disease patients in higher-tier hospitals, and enhances the continuity of their diagnosis and treatment [27]. These dual advances enforce continuity of care and enable consistent health monitoring—core determinants of therapeutic adherence and chronic disease prognosis.

Therefore, we developed hypothesis H1:

**H1.**  *The Policy can improve health outcomes of chronic disease patients*.

The out-of-pocket ratio constitutes a critical determinant of medical financial burden for chronic disease patients, directly influencing both treatment accessibility and ultimate health outcomes. The Policy can systematically reduce this ratio. Firstly, by implementing the “highest-standard integration” principle, the Policy increases reimbursement rates and expands coverage for essential drugs, thereby directly reducing the proportion of medical costs borne by patients [22]. Secondly, the establishment of a unified provincial medical insurance information platform enables seamless cross-regional reimbursement processing, ensuring that chronic disease patients receive standardized benefit settlements regardless of their treatment location. It also effectively eliminates jurisdictional reimbursement disparities that previously inflated out-of-pocket expenditures [28]. Thirdly, the Policy drives innovations in disease-specific payment methods, which integrate diagnosis, treatment, and auxiliary services into fixed-cost packages. This structural reform discourages hospitals from providing unnecessary medical interventions, thereby curbing irrational medical expenditures and reducing patients’ actual financial liabilities [13].

Therefore, we developed hypothesis H2a:

**H2a.**  *The Policy can improve health outcomes of chronic disease patients through decreasing their out-of-pocket ratio*.

Household income is a fundamental economic factor influencing the management of chronic diseases, directly impacting both the accessibility and quality of therapeutic interventions, as well as exerting a critical influence on long-term health outcomes. The Policy enhances household income through two synergistic mechanisms. On one hand, the unification of reimbursement protocols significantly reduces the search costs and commuting expenses that patients previously incurred when seeking medical care in other jurisdictions to obtain higher reimbursement [28]. This liberation of time resources mitigates the compression of working hours, thereby preserving labor income that was previously eroded by fragmented access to healthcare. On the other hand, by reducing the intensity and duration of caregiving labor required for chronic disease patients, the Policy enables the reallocation of the labor force toward productive activities, thereby increasing wage-based earnings. Specifically, the Policy improves the accessibility and convenience of medical care for patients, which in turn reduces the caregiving burden on family members [29]. This alleviation of caregiving demands can liberate productive labor within the household, enabling greater participation in the labor market and increasing earned income.

Therefore, we developed hypothesis H2b:

**H2b.**  *The Policy can improve health outcomes of chronic disease patients through increasing their household income*.

Chronic disease management extends beyond post-onset pharmaceutical interventions to include critical preemptive primary prevention measures, the core of which lies in sustainable lifestyle modifications, achieved through improved dietary quality, regular health monitoring, and increased engagement in physical activity [30]. Elevated household consumption, particularly its structural optimization toward prevention-oriented expenditures, significantly improves chronic disease outcomes. The Policy catalyzes such consumer expenditure through dual channels. Firstly, by reducing families’ financial burden for acute-phase treatment, the Policy redirects financial resources toward preventive health investments [31]. Secondly, the Policy fosters stable expectations regarding medical insurance reimbursement, thereby reducing the precautionary savings that households previously reserved for unexpected medical expenses [31]. This reduction in precautionary savings alleviates credit constraints on consumption loans, enabling households to secure upfront financing for health-enhancing assets (e.g., nutrient-dense foods, fitness memberships). Consequently, the Policy transforms from a mechanism that subsidizes treatment into an enabler of preventive consumption, ultimately benefiting the health outcomes of Chinese chronic disease patients.

Therefore, we developed hypothesis H2c:

**H2c.**  *The Policy can improve health outcomes of chronic disease patients through stimulating their consumer expenditure*.

## 3. Data, Variables, and Model

### 3.1. Data

This study examines the effects of the Policy on health outcomes of chronic disease patients. Information regarding the adoption and implementation timing of the Policy across various provinces in China was manually compiled from publicly available sources. Data pertaining to chronic disease patients were obtained from the China Family Panel Studies (CFPS) database. The CFPS database has conducted biennial surveys since 2010, and has now made its latest dataset from the 2022 survey publicly available. The CFPS database, with its national provincial coverage, longitudinal design, and rich individual-level measures of health status, chronic conditions, and medical insurance, provides the essential structure and variables for this study. Sample selection followed these criteria in line with the research objectives. Firstly, only individuals diagnosed with chronic diseases were included. Secondly, to precisely assess the effects of the Policy, the sample was limited to participants enrolled in the Chinese BMIP. Following the completion of the requisite data cleaning procedures, a total of 26,585 observations were retained, encompassing 31 provinces in China (except Hong Kong, Macao, and Taiwan) over the period from 2010 to 2022.

### 3.2. Variables

#### 3.2.1. Explained Variables

The health outcomes of chronic disease patients constitute the primary variables of interest in this study. Within the academic literature, health outcomes are commonly assessed using clinical indicators and self-rated health (SRH). Clinical indicators—such as blood pressure, body mass index (BMI), and activities of daily living (ADL)—are valued for their objectivity and precision in measurement; however, their collection often entails substantial costs. Moreover, these clinical measures frequently fall short in providing a comprehensive evaluation of an individual’s overall health status and may fail to detect progressive declines in health among chronic disease patients. For instance, diabetic patients may exhibit a phenomenon known as pseudo-stable glycemia, wherein achieved lab targets (e.g., HbA1c or fasting glucose levels) obscure underlying health deterioration. In contrast, SRH captures individuals’ holistic perception, integrating unmeasured biological, psychological, and functional dimensions that often predict long-term health outcomes better than isolated clinical indicators [32]. A seminal study conducted by Idler and Benyamini (1997) [33] demonstrates that SRH serves as a more effective predictor of individual mortality than traditional clinical indicators. Hence, this study utilized SRH as an indicator to assess the health outcomes of chronic disease patients. Within the CFPS database, participants are requested to provide a subjective assessment of their own health status using a 7-point scale. Based on this information, we constructed the variable SRH with values ranging from 1 to 7, where higher scores correspond to better perceived health.

#### 3.2.2. Explanatory Variables

The explanatory variable was the provincial pooling policy of Chinese BMIP (the Policy). This variable was a binary indicator variable coded as 0 or 1, and served to identify whether the Policy had been implemented in the province where the individual resides. If the implementation time was less than or equal to the investigation time, it was assigned a value of 1. Otherwise, it was assigned a value of 0.

#### 3.2.3. Control Variables

Referring to Zhang and Chen (2024) [8], we controlled various individual-level variables and family-level variables. The former included age, gender, household registration, marital status, and internet use, and the latter includednumber of family members and household income per capita.

Table 1 shows the descriptive statistics. The mean SRH score is 3.297, which falls below the midpoint of the scale ranging from 1 to 7. This suggests that SRH values are predominantly distributed toward the lower end of the scale. Consequently, chronic disease patients in China tend to assess their own health status relatively poorly. The mean value of the Policy is 0.079, signifying that the Policy encompasses 7.9% of the sample population. This limited coverage can be attributed to the fact that the Policy in China remains in the pilot phase. These findings align with the results reported by Zhang and Chen (2024) [8], whose study indicated that only 3.7% of individuals in their sample had adopted the Policy.

### 3.3. Model

Following Peng et al. (2025) [22] and Zhang and Chen (2024) [8], this study utilized the difference-in-differences (DID) to test the effects of the Policy on health outcomes of chronic disease patients, as shown in Model 1. The DID method is a widely used approach in policy evaluation and has matured into a robust analytical framework. Its core idea is based on counterfactual inference, which in this study isolated the Policy’s effect by estimating what would have happened to the treatment group in the absence of the Policy. In this study, we applied the DID method to compare changes in the health outcomes of chronic disease patients before and after the implementation of the Policy, as well as between provinces that implemented the Policy and those that did not, in order to identify the Policy’s causal effect.

(1) $${SRH}_{it}=\alpha_{0}+\beta_{0}{Policy}_{it}+\eta C_{it}+u_{t}+v_{jt}+\varepsilon_{it}$$

Here, *i*, *j* and *t* represent individual, county and year, respectively; the coefficient *β* captures how the Policy affects the SRH of Chinese chronic disease patients; *C* represents control variables; *u_t_* represents the year fixed effects (FEs) and *v_jt_* denotes the county × city FE; *α*_0_ represents the intercepted item; and *ε_it_* is the random error vector.

We further employed event-study methodology to assess the parallel trend assumption and verify the absence of significant pre-policy differences.

## 4. Results

### 4.1. Baseline Results

Table 2 presents the outcomes of Model 1. Column (1) is the results without the control variables, while Column (2) shows the results with these variables. Both columns have introduced the fixed effects and the robust standard errors of clustering at the individual level. The coefficient of Policy in Column (1) is 0.459, which suggests that the Policy results in a 0.459-unit increase in the SRH of the treatment group, representing 13.92% of the average SRH (0.459/3.297). Similarly, the corresponding coefficient is 0.300 in Column (2), which means that following the implementation of the Policy, the SRH of the treatment group exhibits an increase of 0.3 units, corresponding to 9.10% of the mean SRH value (0.300/3.297). These findings evidence that the Policy in China has enhanced Chinese chronic disease patients’ health outcomes. Hence, our empirical results support hypothesis H1.

### 4.2. Robustness Tests

#### 4.2.1. Parallel Trend Assumption Assessment

A fundamental requirement for DID to yield an unbiased estimator is the fulfillment of the parallel trend assumption. This assumption stipulates that prior to the implementation of the Policy, there must be no systematic differences in trends between the treatment group and the control group. Following Beck et al. (2010) [34], the event-study methodology was introduced to assess the validity of the parallel trend assumption. The details are shown in Model 2.

(2) $${SRH}_{it}=\alpha_{0}+\sum_{m=-3}^{3}\beta_{0}{Policy}_{it}^{m}+\eta C_{it}+u_{t}+v_{jt}+\varepsilon_{it}$$

**Figure 2:**
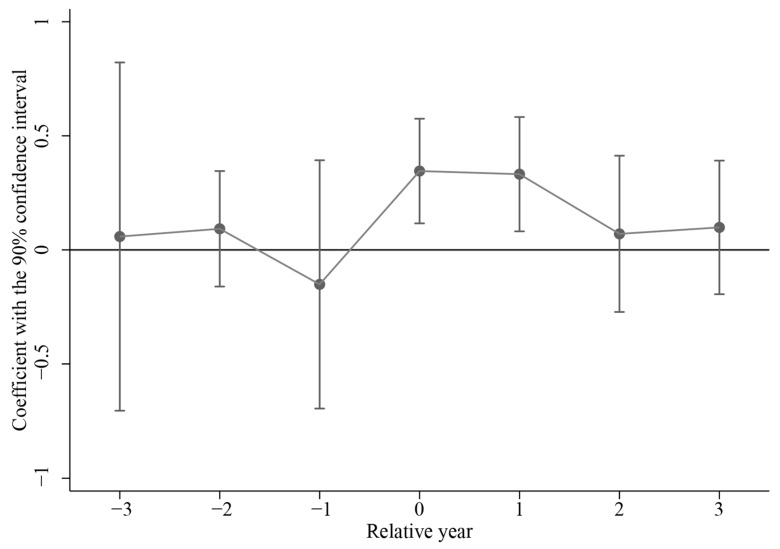
Results of parallel trend hypothesis assessment.

Here, ${Policy}_{it}^{m}$ (*m* < 3) assumes a value of 1 when the difference between the survey year and the implementation year of the Policy is equal to m; ${Policy}_{it}^{3}$ is assigned a value of 1 when that difference is greater than or equal to 3. In all other cases, ${Policy}_{it}^{m}$ (*m* ≤ 3) are assigned a value of 0. To mitigate multicollinearity, instances in which the survey year precedes the implementation year by more than three years were excluded from the model. Figure 2 shows the results of Model 2.

Figure 2 illustrates that the confidence intervals for the three years preceding the implementation of the Policy encompass the zero line, suggesting no statistically significant difference in SRH between the treatment and control groups prior to the intervention of the Policy. In contrast, the confidence intervals for the year of policy implementation and the following year lie entirely above zero, indicating a significant increase in SRH for the treatment group relative to the control group subsequent to the Policy’s enactment. These findings provide robust evidence in support of the parallel trend assumption. Consequently, the DID estimates derived from the benchmark regression in this study can be considered reliable.

#### 4.2.2. PSM-DID

To further address potential sample selection bias between the treatment and control groups, this study employed propensity score matching-DID (PSM-DID) for robustness testing. Specifically, a logistic regression model was employed to estimate the propensity scores, followed by the application of a 1:1 nearest neighbor matching technique for the PSM-DID analysis. The results are presented in Column (1) of Table 3. The regression coefficients and the statistical significance of Policy align with the findings of the benchmark regression, thereby demonstrating the robustness of the aforementioned conclusion.

#### 4.2.3. Alternative Variable Measurement

The Policy manifests in two primary forms. The first is the unified collection and expenditure model, wherein the medical insurance fund is centrally collected, managed, and disbursed at the provincial level. The second form involves a risk adjustment fund model, whereby each city allocates a certain proportion of its fund—typically ranging from 30% to 50%—to be integrated into provincial unified management. This pooled resource is utilized to subsidize regions facing financial deficits, while the municipal funds retain autonomous management rights. It is important to note that the latter form does not constitute strict the Policy, as it lacks comprehensive and uniform management of all municipal medical insurance funds within the province. In the benchmark regression, both forms were initially classified as the Policy. However, in the present analysis, only the former form was recognized as the Policy, with the latter form excluded. Accordingly, the variable Policy*_r_* was reconstructed and regression analysis performed using Model 1. The results, presented in Column (2) of Table 3, align with previous findings, thereby affirming the reliability of the study’s conclusions.

#### 4.2.4. Placebo Tests

**Figure 3:**
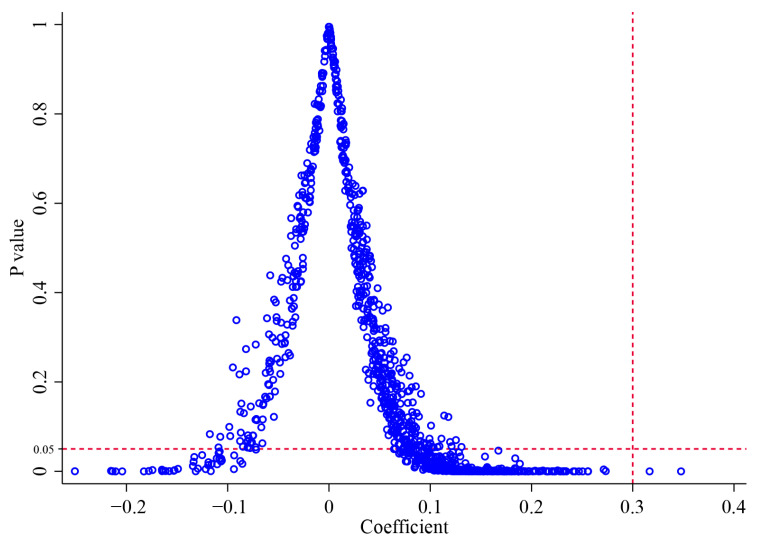
Results of placebo tests.

To exclude the possibility that the benchmark findings were driven by random confounding factors, placebo tests were performed. The fundamental principle involved assessing the likelihood that the benchmark regression results could arise by chance through repeatedly and randomly assigning treatment and control groups. Specifically, each year, provinces were randomly selected (without replacement) from all provinces to match the number of actual policy implementers, forming a hypothetical treatment group. Based on this hypothetical situation, Model 1 was recalculated, and the estimated coefficient of the Policy and its corresponding *p*-value were recorded. Then this procedure was repeated 1000 times, yielding 1000 pairs of coefficients and *p*-values. They are illustrated in Figure 3. It can be seen that the benchmark results represent a low-probability event attributable to random chance (only two simulated situations exhibit a coefficient exceeding 0.300 and a *p*-value below 0.05). Consequently, there is strong evidence to conclude that the Policy has significantly improved health outcomes of chronic disease patients.

### 4.3. Heterogeneity Analysis

#### 4.3.1. Region Heterogeneity

China’s healthcare system exhibits pronounced urban–rural disparities in both the quantity and quality of medical resources. For instance, the number of medical beds per capita in urban areas was 8.02 compared to only 6.52 in rural areas in 2023 (*China Statistical Yearbook 2024*, https://www.stats.gov.cn/sj/ndsj/2024/indexch.htm (accessed on 20 September 2025)). Given that chronic disease management requires continuous, accessible care, such geographical inequities may fundamentally alter the impacts of the Policy on chronic disease patients, necessitating rigorous heterogeneity analysis to uncover divergent health effects of the Policy across regions. This study stratified the overall sample into urban and rural sub-samples according to household registration data. The regression outcomes for urban regions are presented in Column (1) of Table 4, whereas those for rural regions are displayed in Column (2).

The results indicate that the coefficient of Policy is significantly positive in urban contexts, whereas it lacks statistical significance in rural settings. This suggests that the Policy has been effective in enhancing health outcomes of urban chronic disease patients, yet it has not produced a significant impact on those rural patients. In fact, against the backdrop of China’s fragmented healthcare landscape—where rural clinics face critical resource shortages—the Policy has exerted minimal health enhancement for rural chronic disease patients. By contrast, patients with chronic diseases in urban areas have access to more abundant medical resources, higher health literacy, and greater digital literacy. These advantages allow them to utilize medical insurance benefits more effectively, resulting in more significant policy effects within this subgroup.

#### 4.3.2. Income Heterogeneity

The management of chronic diseases is intrinsically sensitive to individuals’ income levels, primarily because of the potentially catastrophic out-of-pocket expenses involved. Based on this, it is plausible that the effects of the Policy vary across different income groups. The sample was divided into two equal groups based on the average per capita household income over the sample period, with income values adjusted for inflation using the gross domestic product (GDP) deflator. Separate regressions were then performed for the low-income group and the high-income group. The results for the former group are presented in Column (3) of Table 4, while those for the latter group are displayed in Column (4).

The coefficients of the Policy in both groups were found to be significantly positive. Notably, the coefficient for the low-income group (0.645) exceeds that of the high-income group (0.123). This difference is statistically significant, as evidenced by a *p* value of 0.006 obtained from Fisher’s permutation test. This divergence stems from the heterogeneous leverage effects of budget constraint mitigation across income strata. By enhancing reimbursement entitlements, the Policy directly reduces patients’ out-of-pocket burden, a financial relief that generates disproportionately larger behavioral responses among low-income groups due to their higher price elasticity of demand for chronic care. Consequently, the marginal health utility derived from medical spending decreases is substantially greater for these individuals, resulting in sharper health gains for low-income groups brought by the Policy.

#### 4.3.3. Education Heterogeneity

The distribution of human capital, especially levels of educational attainment, significantly influences individuals’ health literacy and their ability to effectively utilize health policies. Considering that the Policy involves intricate reimbursement regulations and demands coordinated care management, the resulting health benefits may depend on patients’ proficiency in navigating these complexities. Therefore, it is essential to explicitly investigate how variations in educational attainment affect the efficacy of the Policy.

Individuals without formal education (illiterate or semiliterate) were classified as the low-education group, while those with any formal schooling constituted the high-education group. As presented in Columns (5) and (6) of Table 4, the Policy coefficient is significantly positive only for the high-education cohort (β = 0.172, *p* < 0.05), indicating that the Policy improves health outcomes exclusively among chronic disease patients with educational attainment. Indeed, the high-education patients, owing to their enhanced capacity to interpret health information, effectively integrate resources, and mitigate behavioral biases, have successfully translated policy advantages into concrete health outcomes. Conversely, the low-education individuals may possess inadequate comprehension of the relevant policies, which can result in their failure to obtain eligible medical insurance reimbursements. This limitation consequently impedes the potential health benefits afforded by the Policy.

### 4.4. Underlying Mechanism Analysis

#### 4.4.1. Out-of-Pocket Ratio

The Policy standardizes medical insurance reimbursement regulations across all prefectures belonging to a province. To mitigate institutional resistance during consolidation, the “highest-standard integration” principle (i.e., preferentially adopting the most generous local standard) is institutionalized—deliberately expanding reimbursement scope and rates beyond pre-reform baselines. This design directly reduces out-of-pocket expenditures for chronic disease patients, thereby decreasing their out-of-pocket ratio and enabling health improvements. Accordingly, Model 3 was developed to empirically examine this mechanism, as outlined below.

(3) $${Ratio}_{it}=\alpha_{0}+\beta_{1}{Policy}_{it}+\eta C_{it}+u_{t}+v_{jt}+\varepsilon_{it}$$

Here, *Ratio* represents out-of-pocket ratio, which is quantified by the ratio of out-of-pocket expenditures to the overall medical costs.

Table 5, Column (1) presents the results, which show that the coefficient of Policy is significant and negative. This finding suggests that the Policy decreases the out-of-pocket ratio experienced by chronic disease patients, thereby providing empirical support for hypothesis H2a.

#### 4.4.2. Household Income

The Policy has strengthened Chinese medical insurance benefits for chronic disease patients, facilitating improved recovery of their productivity, shortening periods of work absenteeism, and increasing participation in the labor market. Consequently, this leads to increased household income and offers economic support that contributes to improved health outcomes for chronic disease patients. Hence, Model 4 was introduced to test this mechanism.

(4) $${Income}_{it}=\alpha_{0}+\beta_{2}{Policy}_{it}+\eta C_{it}+u_{t}+v_{jt}+\varepsilon_{it}$$

Here, *Income* represents household income, which was measured by the logarithm of the total household income divided by the number of household members.

The coefficient of Policy in Table 5, Column (2) shows a significant and positive value. This evidences that the Policy increases the household income of Chinese chronic disease patients, thereby confirming hypothesis H2b.

#### 4.4.3. Consumer Expenditure

By enhancing benefit entitlements under China’s BMIP, the Policy reduces precautionary savings for chronic disease management and improves patients’ financial security. This facilitates a reallocation of household budgets toward current consumption, synergistically enhancing both quality of life and health outcomes. Hence, Model 5 was constructed to test this mechanism.

(5) $${Expenditure}_{it}=\alpha_{0}+\beta_{3}{Policy}_{it}+\eta C_{it}+u_{t}+v_{jt}+\varepsilon_{it}$$

Here, *Expenditure* represents consumer expenditure, which was measured by the logarithm of total household consumption divided by the number of household members.

Column (3) of Table 5 presents the results for the consumer expenditure mechanism. The coefficient of Policy is significantly positive at the 1% level, which means that the Policy increases the per capita consumption expenditure of families with chronic disease patients. These results support hypothesis H2c.

### 4.5. Further Analysis: The Role of Digital Technology

The Policy entails a comprehensive provincial integration of Chinese medical insurance, which has unintentionally increased the complexity and costs associated with medical governance [12]. This is demonstrated by coordination deficiencies among diverse healthcare stakeholders, suboptimal integration of medical data across different regions, and an increasing need for more accurate predictive and monitoring technologies. Chronic disease governance constitutes a multifaceted and systematic endeavor that necessitates ongoing monitoring of physiological functions, sustained behavioral modifications in lifestyle, and the administration of targeted pharmacological treatments [30]. Crucially, the effective management of medical care and chronic diseases necessitates the implementation of digital technologies such as big data, artificial intelligence, and the Internet of Things [35,36]. The digital technologies discussed in this study encompass information and communication technologies specifically applied in the healthcare sector for the collection, transmission, analysis, and application of data. These include but are not limited to integrated provincial information platforms, telemedicine and remote consultation systems, artificial intelligence and big data analytics, the Internet of Things and wearable devices, and mobile health applications. It is posited that digital technologies function not only as operational instruments but also as fundamental governance frameworks transforming the landscape of Chinese medical care and health management [37]. Thus, interrogating the role of digital technology in how the Policy affects Chinese chronic disease patients’ health outcomes is not only academically salient but existentially imperative.

This study developed Model 6 to examine the moderating role of digital technology in enhancing the health outcomes of chronic disease patients due to the Policy.

(6) $${SRH}_{it}=\alpha_{0}+\beta {Policy}_{it}+\lambda {Digital}_{ikt}+\theta {Policy}_{it}\times {Digital}_{ikt}+\eta C_{it}+u_{t}+v_{jt}+\varepsilon_{it}$$

Here, *k* indicates province and Digital represents digital technology. Digital technology was measured from both the input and output perspectives, respectively. Digital technology input (Input) was quantified by aggregated investments in digital technology research and development (R&D) from governmental and corporate sources, while digital technology output (Output) was measured using the total number of internet broadband access ports, landline telephone subscribers, and mobile phone users. They were both calculated at the provincial level, and both metrics underwent normalization through division by provincial GDP to mitigate scale effects.

Table 6 presents the results of Model 6, where Column (1) presents the results of Input and Column (2) presents the results of Output. As shown, the coefficients of both Policy × Input and Policy × Output are significant and positive at the 5% level. These findings indicate that digital technology has significantly intensified the enhancement of the Policy on chronic disease patients’ health outcomes.

## 5. Discussion

### 5.1. How the Policy Results in Better Health: Three Key Channels

The results evidence the Policy enhances Chinese chronic disease patients’ health outcome across various robustness assessments. The Policy can enhance chronic disease patients’ health outcomes by reducing the out-of-pocket ratio, increasing household income, and stimulating consumer expenditure. Firstly, such a reduction in out-of-pocket ratio facilitates increases utilization of healthcare services and mitigates the psychological stress associated with medical costs, ultimately contributing to improvement in patients’ overall health outcomes. Secondly, the increase in household income can enhance the ability of chronic disease patients to afford medical services and medication, thereby facilitating improvements in their health outcomes. Thirdly, increased household consumer expenditures, specifically those on health management, nutritional quality, and physical activity, synergistically enhance chronic disease treatment efficacy, driving measurable improvements in patients’ health outcomes.

### 5.2. Unlocking Policy Potential: How Digital Technology Acts as an Amplifier

This study evidences that digital technology can amplify the effectiveness of the Policy in Chinese chronic disease patients’ health outcomes. This phenomenon may be attributed to the following factors. Firstly, digital technology can reconfigure the spatiotemporal allocation of medical resources [38]. Although the Policy has achieved provincial-level consolidation of the medical insurance fund, enabling patients with chronic diseases to access cross-regional healthcare services within the province, they continue to face considerable logistical and financial obstacles. The commuting expenses involved in traveling across regions substantially limit patients’ actual use of such services. In this context, digital technologies—such as telemedicine and remote consultation platforms—mitigate the constraints of geographical distance by compressing time and space [39], thereby reducing cost-related barriers and significantly enhancing the Policy’s effectiveness in improving health outcomes among chronic disease patients.

Secondly, digital technology can elevate the service quality of medical institutions. Digital technology has greatly improved information sharing and collaboration among medical institutions, leading to more standardized and consistent healthcare practices. For instance, artificial intelligence can assist in chronic disease screening, prognosis assessment, and treatment planning [40]. Moreover, digital progress facilitates interoperability between healthcare organizations by supporting mutual recognition of diagnostic results and integration of clinical management strategies [40].

Thirdly, digital technology can reengineer the operational architecture of insurance governance [41]. Under the Policy, oversight of medical insurance has been elevated from the municipal to the provincial level, which may create opportunities for moral hazard from both healthcare providers and patients. Digital technology strengthens the capacity of provincial authorities to administer medical insurance funds efficiently and reduce unnecessary financial losses [42]. The evolution of digital tools has accelerated the establishment of province-wide medical insurance information platforms, enhanced intelligent monitoring of healthcare services, reduced instances of overdiagnosis and overtreatment, and decreased out-of-pocket expenses for chronic disease patients. Additionally, it has simplified the process of cross-regional insurance reimbursement and increased the number of eligible reimbursements for these patients.

Fourthly, digital technology can cultivate the autonomous health management capabilities of patients. Digital technology also helps lower knowledge and behavioral barriers that hinder effective self-management of health [17]. For example, wearable medical devices enable continuous disease monitoring and support treatment adherence among people with chronic conditions, while algorithm-driven personalized nutrition and exercise plans improve the efficacy of lifestyle interventions. By strengthening patients’ self-management capabilities, these technologies amplify the health-promoting effects of the Policy.

Fifthly, by leveraging in-depth analysis of the large-scale data aggregated through provincial pooling, digital technology supports the design of more efficient medical insurance strategies [17]. It enables governments to monitor regional prevalence of chronic diseases and anticipate patient healthcare needs, thereby improving the mobilization and allocation of medical resources. Through big data analytics, digital technology can also provide personalized health assessments and tailored treatment plans for individuals with chronic diseases, substantially boosting their health outcomes.

### 5.3. Contributions

The main contributions are as follows. Firstly, this study investigated the Policy’s health impacts with a focus on chronic diseases. Although certain studies have investigated health effects of the Policy, none has yet examined it from the perspective of chronic diseases. This study integrated chronic diseases into the analytical framework, advancing research on provincial pooling of medical insurance and enriching the understanding of its role in chronic disease management. On one hand, chronic diseases have become a major determinant of population health in China, making it essential to examine the influence of provincial pooling, a pivotal medical insurance reform of China, on chronic disease outcomes. On the other hand, the long-term and systematic nature of chronic disease management necessitates effective reductions in household medical burdens and greater integration of medical resources. Therefore, the chronic disease perspective offers a relevant lens through which to assess health effects of the Policy, thereby enabling a rigorous evaluation of the reform’s effectiveness.

Secondly, this study identified several mechanisms through which the Policy enhances health outcomes of chronic disease patients. Existing research has primarily examined health effects of the Policy through the lens of reduced medical burdens, yet limited attention has been given to household income and consumer expenditure mechanisms. In reality, prevention and screening play critical roles in chronic disease management. Adopting health-promoting behaviors, improving nutritional quality, and undergoing regular check-ups can significantly reduce the incidence of chronic conditions and enhance treatment efficacy. Thus, analyzing this Policy’s impact on health outcomes from the perspective of household economic mechanisms remains an essential and understudied avenue.

Thirdly, this study examined the amplifying role of digital technology in enhancing the health-related benefits of the Policy. Existing research has largely overlooked the role of digital technology, which could lead to a misinterpretation of the Policy’s health impacts. On the one hand, provincial pooling introduces greater complexity in medical insurance administration due to unified policy management across regions. Digital technology can mitigate this by optimizing operational efficiency, thereby strengthening policy efficacy. On the other hand, chronic diseases require systematic long-term management. Digital technology facilitates the flow of medical services to underserved areas, enabling better integration of regional healthcare resources. Furthermore, digital technology empowers patients by enhancing health literacy and self-management capabilities, thereby improving chronic disease outcomes.

### 5.4. Limitations and Future Research Directions

This study has several limitations that warrant attention in future research. Firstly, the use of SRH may not fully capture the actual health status of individuals with chronic diseases. As a subjective measure, SRH is inherently susceptible to biases such as social desirability and recall bias, which can lead to intentional or unintentional misreporting. Should clinical indicator data become available, revisiting this research question with objective health measures could yield complementary insights. Secondly, the measurement of digital technology use in this study could be further refined. Although both input- and output-side indicators were employed, the analysis did not examine specific healthcare applications, such as the adoption and impact of telemedicine. Future studies supported by more granular and context-specific data should incorporate more precise metrics to enhance the depth and accuracy of understanding in this area.

Future research directions include the following. In terms of methodology, qualitative approaches can be employed to investigate specific issues, such as using interviews and surveys to systematically examine the strengths and limitations of both high-performing and underperforming regions that have implemented the Policy, in order to generate targeted and actionable insights. Regarding research content, the analysis of underlying mechanisms can be further deepened, shifting from a descriptive understanding of “what the effect is” to a more nuanced exploration of “how it works in practice.” Research could, for instance, examine whether different types of digital technologies lead to significant variations in their policy amplification effects. From a research perspective, the scope can be broadened from a China-specific focus to international comparative studies. One could, for example, investigate the distinctive characteristics and evolution of integrated healthcare insurance systems in developing versus developed countries. Future research can also extract transferable lessons from China’s experience to enhance chronic disease management in low- and middle-income countries (LMICs), emphasizing centralized funding, digital infrastructure, and equity-oriented measures.

### 5.5. Policy Implications

First, the government should accelerate the nationwide implementation of the Policy. Provincial pooling allows for better coordination in the allocation and use of medical insurance funds across broader regions, which enhances the risk resilience of the funds and offers greater flexibility to improve reimbursement levels and benefits for enrollees. At the same time, efforts should be made to transition from mere “formal uniformity” to authentic “substantive equity” within the provincial pooling framework. It is essential to move away from one-size-fits-all policies and instead prioritize substantive fairness. This could include providing targeted subsidies and medical vouchers to low-income and rural populations to reduce their financial healthcare burdens. In addition, chronic diseases could be classified into different tiers, such as basic (e.g., hypertension, diabetes), intermediate (e.g., coronary heart disease), and special (e.g., rare diseases), to facilitate the design of differentiated insurance policies tailored to the specific needs of each category.

Second, the government should emphasize the integration of digital technologies into healthcare to enhance the efficacy of the Policy. A key step is to establish a cross-regional, cross-departmental medical data sharing platform that integrates the entire healthcare process, thus simplifying medical visits, improving disease prediction, optimizing insurance reimbursement, and strengthening fund oversight. Increased support should also be given to artificial intelligence-assisted diagnostics and telemedicine to help channel high-quality medical resources toward rural areas and primary care institutions. This would help narrow the urban–rural health resource gap and encourage a more regionally balanced distribution of healthcare services. Furthermore, initiatives such as “digital health literacy” programs should be introduced to help patients with chronic diseases better use digital tools. These could involve inclusive digital skills training, government-subsidized digital products and wearable devices, and the development of intuitive, age-friendly artificial intelligence-assisted diagnostic systems and interactive health management applications.

Third, the government should refine the causal pathways through which the Policy improves health outcomes for chronic disease patients. It is crucial to further reduce financial burdens by expanding insurance coverage for outpatient chronic care, improving the centralized drug procurement mechanism, and developing new payment models specific to chronic diseases. Policies should also promote vocational rehabilitation programs to help patients increase their labor income and should introduce legal protections against workplace discrimination. In addition, efforts are needed to unlock consumption potential currently limited by medical expenses, encouraging greater investment in preventive care, early screening, and nutritional interventions. Given the long-term nature of chronic diseases, such integrated measures can significantly improve both the effectiveness and sustainability of chronic disease management.

## 6. Conclusions

Amid rapid industrialization, urbanization, and population aging in China, chronic diseases have surged in prevalence and mortality, emerging as a major public health challenge characterized by long-term progression, high burden, poor control, and a growing younger patient base. The provincial pooling policy of the Chinese BMIP has optimized resource allocation, enhanced patient rights, and strengthened healthcare management, which creates opportunities to improve chronic disease patients’ health outcomes. Concurrently, digital technology can boost information efficiency and thus amplify the Policy’s effects. This study therefore analyzed how the Policy shapes health outcomes in chronic disease patients, with digital technology acting as an amplifier that modifies the Policy’s effectiveness, utilizing a total of 26,585 observations that encompassed 31 provinces in China (except Hong Kong, Macao, and Taiwan) over the period from 2010 to 2022.

The results evidence that the Policy enhances Chinese chronic disease patients’ health outcomes across various robustness assessments. However, the effects exhibit heterogeneity, where the Policy can more effectively improve the health outcomes of urban patients, low-income patients, and highly educated patients. The mechanism analysis indicates that the Policy can enhance chronic disease patients’ health outcomes by reducing the out-of-pocket ratio, increasing household income, and stimulating consumer expenditure. Furthermore, digital technology can amplify the effectiveness of the Policy in Chinese chronic disease patients’ health outcomes.

Based on our findings, we propose three actionable policy priorities. First, nationwide rollout of the Policy should be accelerated with deliberate attention to equity using targeted subsidies and tiered disease management protocols to ensure benefits reach vulnerable populations. Second, digital integration must become a cornerstone of healthcare delivery, leveraging unified data platforms, artificial intelligence-assisted diagnostics, and digital literacy initiatives to bridge urban–rural divides and enhance system-wide efficiency. Finally, policy refinement should focus on strengthening implementation: expanding chronic disease coverage, supporting vocational reintegration of patients, and incentivizing preventive care through financial mechanisms.

## Figures and Tables

**Table 1:** Descriptive statistics.

Variables	N	Mean	S.D.	Min	Max
	(1)	(2)	(3)	(4)	(5)
**Core variables**					
SRH (range from 1 to 7; higher self-assessment means better overall health)	26,585	3.297	1.841	1	7
Policy (have implemented the Policy = 1; not yet = 0)	26,585	0.079	0.270	0	1
**Control variables**					
Age	26,585	55.718	14.073	16	95
Gender (male = 1; female = 0)	26,585	0.440	0.496	0	1
Household registration (urban = 1; rural = 0)	26,585	0.458	0.498	0	1
Marital status (married = 1; others = 0)	26,585	0.852	0.355	0	1
Internet use (yes = 1; no = 0)	26,585	0.206	0.404	0	1
Number of family members	26,585	4.067	1.955	1	17
Household income per capita (in log, RMB)	26,585	9.007	1.917	0	15.243

**Table 2:** Baseline regression results.

	(1)	(2)
Policy	0.459 ***	0.300 ***
	(0.082)	(0.076)
Controls		√
Year FE	√	√
County × Year FE	√	√
N	26,585	26,585
R^2^	0.614	0.634

*** *p* < 0.01. The values in parentheses represent the robust standard errors of clustering at the individual level.

**Table 3:** Robustness test results.

	PSM-DID (1)	Alternative Measurement of Policy (2)
Policy	0.300 ***	
	(0.085)	
Policy		0.299 ***
		(0.074)
Controls	√	√
Year FE	√	√
County × Year FE	√	√
N	6229	26,585
R^2^	0.712	0.634

*** *p* < 0.01. The values in parentheses represent the robust standard errors of clustering at the individual level.

**Table 4:** Heterogeneity analysis results.

	Region Heterogeneity		Income Heterogeneity		Education Heterogeneity	
	Urban	Rural	Low	High	Low	High
	(1)	(2)	(3)	(4)	(5)	(6)
Policy	0.311 ***	0.204	0.645 ***	0.192 **	0.404	0.172 **
	(0.086)	(0.183)	(0.235)	(0.088)	(0.324)	(0.084)
Controls	√	√	√	√	√	√
Year FE	√	√	√	√	√	√
County × Year FE	√	√	√	√	√	√
N	12,179	14,406	12,279	14,306	9198	17,387
R^2^	0.692	0.626	0.644	0.653	0.615	0.674

*** *p* < 0.01; ** *p* < 0.05. The values in parentheses represent the robust standard errors of clustering at the individual level. The coefficient difference between the low-income group and high-income group is statistically significant, as evidenced by a *p* value of 0.006 obtained from Fisher’s permutation test.

**Table 5:** Underlying mechanism results.

	Medical Burden (1)	Household Income (2)	Consumer Expenditure (3)
Policy	−0.042 **	0.438 ***	0.128 ***
	(0.020)	(0.168)	(0.049)
Controls	√	√	√
Year FE	√	√	√
County × Year FE	√	√	√
N	23,664	26,585	24,412
R^2^	0.452	0.303	0.493

*** *p* < 0.01; ** *p* < 0.05. The values in parentheses represent the robust standard errors of clustering at the individual level. Samples in which out-of-pocket medical expenses exceed total medical expenses are excluded from Column (1). Additionally, samples with zero consumer expenditure are excluded from Column (3).

**Table 6:** Results on the role of digital technology.

	Digital Technology Input	Digital Technology Output
	(1)	(2)
Policy	−0.329	1.047 ***
	(0.308)	(0.382)
Input	−0.002	
	(0.031)	
Policy × Input	0.053 **	
	(0.026)	
Output		2.673
		(2.632)
Policy × Output		6.894 **
		(3.475)
Controls	√	√
Year FE	√	√
County × Year FE	√	√
N	26,585	26,585
R^2^	0.634	0.634

*** *p* < 0.01; ** *p* < 0.05. The values in parentheses represent the robust standard errors of clustering at the individual level.

## Data Availability

The original data are available at https://www.isss.pku.edu.cn/cfps/index.htm (accessed on 14 June 2025).
